# Predictive value of vascular endothelial growth factor (VEGF) in metastasis and prognosis of human colorectal cancer.

**DOI:** 10.1038/bjc.1998.688

**Published:** 1998-11

**Authors:** S. I. Ishigami, S. Arii, M. Furutani, M. Niwano, T. Harada, M. Mizumoto, A. Mori, H. Onodera, M. Imamura

**Affiliations:** First Department of Surgery, Faculty of Medicine, Kyoto University, Japan.

## Abstract

**Images:**


British Journal of Cancer (1998) 78(10), 1379–1384
© 1998 Cancer Research Campaign

### Predictive value of vascular endothelial growth factor (VEGF) in metastasis and prognosis of human colorectal cancer

**S-I Ishigami, S Arii, M Furutani, M Niwano, T Harada, M Mizumoto, A Mori, H Onodera and M Imamura**

First Department of Surgery, Faculty of Medicine, Kyoto University, Kyoto, Japan

**Summary** Vascular endothelial growth factor (VEGF) may affect the phenotype of cancer cells, such as growth velocity and metastatic potential, due to its probable multifunctional property including a mitogenic activity for vascular endothelial cells. The present study was designed to investigate the association of VEGF mRNA expression with progression and metastasis of human colorectal cancer. The level of VEGF mRNA expression was quantified by Northern blot hybridization in tumorous and non-tumorous tissues obtained from 60 primary colorectal cancer patients. The ratio of the former to the latter was defined as the VEGF T/N ratio, and the prognostic significance of this ratio, following surgery, in addition to the relationship to progression and metastatic potential, was evaluated. The value of the VEGF T/N ratio was significantly correlated with the depth of tumour infiltration ($P = 0.046$), the incidence of liver metastasis ($P < 0.0001$) and lymph node metastasis ($P = 0.036$). Patient prognosis was estimated by the Kaplan–Meier method and the log-rank test. When the VEGF T/N ratio was higher than 4.8 for which the $\chi^2$ value of the log-rank test was maximal, the tumour was defined as showing overexpression of VEGF mRNA. Patients with overexpression of VEGF mRNA demonstrated poorer survival than patients without overexpression of VEGF mRNA ($P < 0.001$). The overall estimated hazard ratio for death in patients with overexpression of VEGF mRNA was 1.94 according to a multivariate analysis ($P = 0.005$). Thus, VEGF is associated with the progression, invasion and metastasis of colorectal cancer, and overexpression of VEGF mRNA in the primary tumour is assumed to be closely correlated with poor prognosis in colorectal cancer patients. Moreover, the VEGF T/N ratio may be used as an independent prognostic marker in colorectal cancer patients.

**Keywords:** colorectal cancer; the vascular endothelial growth factor tumorous/non-tumorous tissue ratio; overexpression of vascular endothelial growth factor mRNA; metastatic potential; Cox's proportional hazard model

Vascular endothelial growth factor (VEGF) was discovered in 1983, and first called vascular permeability factor (VPF) (Senger et al, 1983). In 1989, a growth factor specific for vascular endothelial cells was purified form the media conditioned by bovine pituitary follicular stellate cells, and this factor was named VEGF (Ferrara et al, 1989).

Recent studies have demonstrated that VEGF is strongly expressed in several human solid tumours, and its expression is correlated with the density of microvessels in tumours of the kidney (Takahashi et al, 1994), breast (Toi et al, 1994), brain (Berkman et al, 1993; Samoto et al, 1995), colon (Takahashi et al, 1995; Takahashi et al, 1997), stomach (Maeda et al, 1996), lung (Mattern et al, 1996), oesophagus (Inoue et al, 1997) and liver (Mise et al, 1996). Concerning VEGF expression in colorectal cancer tissues, Brown et al (1993) first described that the malignant epithelial cells strongly expressed VEGF mRNA in contrast to normal epithelium by in situ hybridization, and that tumour cells stained strongly for VEGF protein by immunohistochemistry (Brown et al, 1993). Furthermore, experimental studies have shown that tumours with high expression of VEGF grew rapidly (Ferrara et al, 1993; Kondo et al, 1993) and exhibited metastatic ability (Asano et al, 1995; Warren et al, 1995). However, very few clinical investigations have been reported regarding the correlation between VEGF and the progression of colorectal cancer. Takahashi et al (1995) showed that VEGF expression at protein level detected by immunohistochemistry was correlated with vessel count in the tumour and metastasis, and may be useful for predicting distant recurrence in patients with node-negative colon cancer (Takahashi et al, 1997). However, they did not make clear the correlation between VEGF expression and hepatic or lymph node metastasis.

In the present study, we directed our attention to the relationship among VEGF mRNA expression with Northern blot analysis, clinicopathological variables, including hepatic and lymph node metastasis, and survival with prospective study.

Northern blot analysis is thought to provide a more objective quantification than immunohistochemical study and we preferred this method to immunohistochemistry. Moreover, considering that biological activities of VEGF are not merely induction of angiogenesis (Gabrilovich et al, 1996; Tsurumi et al, 1997; van der Zee et al, 1997), we focused on VEGF mRNA expression itself without studying the correlation with number of vessels in the tumour.

Evidence will be presented indicating that VEGF mRNA expression plays a predictive role in the prognosis and the hepatic and lymph node metastasis of colorectal cancer patients.

*Received 29 September 1997*
*Revised 2 April 1998*
*Accepted 7 April 1998*

*Correspondence to:* S-I Ishigami, Otsu Red Cross Hospital, 1-1-35 Nagara, Otsu City, Siga, 520–8511, Japan

**Table 1** Correlations between VEGF mRNA expression and clinicopathological parameters

| Parameter | Patients No. | Patients % | VEGF expression Mean ± s.d. | VEGF expression Range | t-test[a] | P-value[b] |
|---|---|---|---|---|---|---|
| Sex | | | | | | |
| Male | 40 | 66.7 | 4.74 ± 5.02 | 0.57–23.85 | | |
| Female | 20 | 33.3 | 2.59 ± 3.91 | 0.67–18.63 | 1.680 | NS (0.100) |
| Age (years) | | | | | | |
| ≤60 | 18 | 30.0 | 3.59 ± 4.20 | 0.69–18.63 | | |
| >60 | 42 | 70.0 | 4.21 ± 5.02 | 0.57–23.85 | 0.461 | NS (0.646) |
| Tumour location | | | | | | |
| Colon | 31 | 51.7 | 3.82 ± 4.12 | 0.57–20.58 | | |
| Rectum | 29 | 48.3 | 4.24 ± 5.42 | 0.69–23.85 | 0.339 | NS (0.736) |
| Tumour size (cm) | | | | | | |
| ≤ 5.0 | 30 | 50.0 | 3.46 ± 3.96 | 0.57–20.58 | | |
| > 5.0 | 30 | 50.0 | 4.59 ± 5.45 | 0.84–23.85 | 0.914 | NS (0.365) |
| Depth of infiltration | | | | | | |
| ≤pm[c] | 17 | 28.3 | 2.08 ± 1.29 | 0.57–4.57 | | |
| >pm | 43 | 71.7 | 4.79 ± 5.39 | 0.69–23.85 | 2.043 | 0.046 |
| Tumour differentiation | | | | | | |
| Well | 19 | 31.7 | 2.92 ± 2.72 | 0.57–12.07 | | |
| Moderate | 41 | 68.3 | 4.54 ± 5.40 | 0.67–23.85 | 1.228 | NS (0.224) |
| Lymphatic involvement | | | | | | |
| ly (–) | 3 | 5.0 | 2.01 ± 0.32 | 1.38–2.44 | | |
| ly (+) | 57 | 95.0 | 4.13 ± 23.60 | 0.57–23.85 | 0.749 | NS (0.457) |
| Vascular involvement | | | | | | |
| v (–) | 9 | 15.0 | 1.73 ± 0.22 | 0.57–2.44 | | |
| v (+) | 51 | 85.0 | 4.43 ± 25.52 | 1.03–23.85 | 1.589 | NS (0.118) |
| Liver metastasis (synchronous) | | | | | | |
| H (–) | 45 | 75.0 | 2.57 ± 1.98 | 0.57–8.68 | | |
| H (+) | 15 | 25.0 | 8.39 ± 7.48 | 1.03–23.85 | 4.813 | $P < 0.0001$ |
| Lymph node metastasis | | | | | | |
| N (–) | 27 | 45.0 | 2.61 ± 3.75 | 0.57–20.58 | | |
| N (+) | 33 | 55.0 | 5.18 ± 5.22 | 0.69–23.85 | 2.142 | 0.036 |
| Dukes' stage | | | | | | |
| A | 13 | 21.7 | 2.10 ± 1.25 | 0.57–4.57 | | 0.019[d] |
| B | 10 | 16.7 | 1.86 ± 1.01 | 0.94–3.68 | | 0.018[d] |
| C | 19 | 31.7 | 3.12 ± 2.54 | 0.69–8.68 | | 0.015[d] |
| D | 18 | 30.0 | 7.57 ± 7.09 | 1.03–23.85 | | |

[a]Unpaired t-test was used for comparison of two groups. [b]A two-tailed P-value ≤ 0.05 was considered to indicate statistical significance. [c]Tumour infiltration is confined to the proprial muscularis layer of the mucosa. [d]The P-value against Dukes' D cases. NS, not significant.

### MATERIALS AND METHODS

### Human samples and clinicopathological data

Sixty primary colorectal cancer specimens were obtained from 60 patients (40 men and 20 women; age range, 43–90 years; mean age 65.3 years) who had undergone surgery from April 1994 to July 1996 in the First Department of Surgery, Faculty of Medicine, at the Kyoto University Hospital. Patients' profiles are listed in Table 1. Except Dukes' A cases, these patients were uniformly given post-operative adjuvant chemotherapy with oral administration of anti-cancer agents of fluorouracil type. None of the patients received irradiation or chemotherapy before surgery.

After the removal of necrotic tissue, tumorous tissue and adjacent normal mucosa without the underlying muscularis layer were collected. Whole tumorous tissue was divided into two pieces. One specimen was frozen immediately and stored at –80°C until processing, and the other was fixed for the purpose of histopathological examination. After checking the distribution of cancer cells and stromal cells, RNA extraction was performed. Consequently, there was no apparent difference in their distribution among all specimens, probably because of the similar grade of differentiation of cancers. The clinical features of these patients were noted with reference to clinical reports and pathology reports, including Dukes' clinical classification, histological type, liver metastasis, lymph node metastasis, lymphatic involvement and vascular involvement of the disease. Haematoxylin and eosin (H&E) staining was routinely performed to determine histological type, lymphatic involvement and vascular involvement in all cases. In 21 cases in which lymphatic involvement or vascular involvement was diagnosed to be negative by H&E staining, Elastica Van Gieson staining was performed to revaluate them.

### Preparation of RNA from human samples

Guanidine isothiocyanate (GTC) and caesium chloride were purchased from Wako Pure Chemical Industries (Japan). Oligotex-dT30(Super) was purchased from JSR Corporation (Japan). Tissue specimens were crushed in liquid nitrogen, and homogenized with 4 M GTC containing 25 mM sodium citrate (pH 7.0), 0.5% lauroyl sarcosine and 0.1 M β-mercaptoethanol. The tissue homogenate was then layered over 5.7 M caesium chloride containing 0.01 M disodium EDTA (pH 7.0), and ultracentrifuged at 180 000 *g* at 20°C for 15–18 h. Pellets, which were located at the bottom of the centrifugation tubes, were collected and total cytoplasmic RNA was extracted using the phenol–chloroform method (Sambrook et al, 1989). Poly(A)$^+$ RNA was purified from total RNA using Oligotex-dT30(Super) according to the method described previously (Kuribayashi et al, 1988).

### DNA probes for Northern blot analysis

The DNA probe for human $VEGF_{165}$ protein was prepared as follows: 5 µg of human liver total RNA was reverse transcribed with random primers, using a commercial kit (First Strand Synthesis Kit; Pharmacia, Piscataway, NJ, USA). The resulting complementary DNA (cDNA) mixture was subjected to 30 cycles (1 min at 94°C, 1 min at 55°C and 1 min at 72°C) of polymerase chain reaction (PCR) amplification using a DNA thermal cycler (Aster, Japan). *Taq* DNA polymerase (Toyobo, Japan) and specific VEGF primers. The following oligonucleotide primers, which were based on the human VEGF cDNA sequence, were used (Tischer et al, 1991): 'sense primer', 5′-TTGCTGCTCTAC-CTCCAC-3′; 'antisense primer', 5′-AATGCTTTCTCCGCTCTG-3′. Two kinds of PCR products, consisting of 418 bp and 490 bp, encoding $VEGF_{165}$ and $VEGF_{189}$, respectively, were obtained. The PCR product that encoded $VEGF_{165}$ was cloned into the *Eco*RV site of the pBluescript SK(–) plasmid, and the insert was confirmed by sequencing (Marchuk et al, 1991). The insert was purified and used as a probe in Northern blot analysis.

### Northern blot analysis

Hybond-N$^+$ nylon membrane, Rapid-hybridization buffer and the Megaprime DNA labelling kit were purchased from Amersham

 © Cancer Research Campaign 1998

**Figure 1** VEGF mRNA expression in colorectal cancer. Poly-A⁺ RNA (5 μg) was electrophoresed on a 1.0% agarose gel, and then transferred to a Hybond-N⁺ nylon membrane. Blotted membranes were hybridized using [α-$^{32}$P]dCTP-labelled human VEGF165 cDNA, and the same membrane was rehybridized using S26 ribosomal protein cDNA. The expression of 3.7-kb VEGF mRNA transcript was detected in both tumorous tissue and adjacent normal mucosa. N, normal colon mucosa; T, tumour; S26rp, S26 ribosomal protein

Lifescience (UK). [α-$^{32}$P]dCTP was purchased from ICN Biomedicals (USA). Poly-A⁺ mRNA (5 μg) was electrophoresed on a 1.0% agarose gel containing 2.2 M formaldehyde in 1 × 3 (N-morpholino) propanesulphonic acid (MOPS) buffer (20 mM MOPS, 5 mM sodium acetate, 1 mM EDTA-2Na], and then transferred to a Hybond-N⁺ nylon membrane by capillary blotting, followed by ultraviolet (UV) cross-linkage. Blotted membranes were prehybridized in Rapid-hybridization buffer at 65°C for 30 min. [α-$^{32}$P]dCTP-labelled human $VEGF_{165}$ cDNA was prepared using a Megaprime DNA labelling kit. If the radioactivity in the reaction buffer was high, a Sephadex-G column was used to remove free radioisotopes. Hybridization was performed at 65°C for 2 h and then the membranes were washed twice for 10 min at room temperature in 2 × standard saline citrate (SSC) (300 mM sodium chloride, 30 mM sodium citrate) with 0.1% sodium dodecyl sulphate (SDS). When background radioactivity was high, membranes were washed again at 65°C for 20 min in 1 × SSC with 0.1% SDS, and then at 65°C for 15 min in 0.1 × SSC with 0.1% SDS. Membranes were exposed to Konica X-ray films at −80°C for appropriate intervals. The membranes were rehybridized with S26 ribosomal protein (S26rp) DNA probe as an internal control. All techniques were performed according to standard methods described previously (Sambrook et al, 1989). The level of VEGF mRNA expression was quantified by densitometric analysis using Densitograph, Ver.4.0 (ATTO, Japan) in tumorous and non-tumorous tissues, and was normalized to S26rp mRNA expression. The ratio of the former to the latter was defined as the VEGF T/N ratio, and the prognostic significance after surgery and the relationship to metastatic potential were evaluated.

**Figure 2** Kaplan–Meier overall survival curves of 56 colorectal cancer patients after surgery. When the cut-off point of the VEGF T/N ratio ranged from 2.8 to 12.0, the difference between survivors and non-survivors was statistically significant. To determine the optimal cut-off point of the VEGF T/N ratio, the analytical method based on which $\chi^2$ value of the log-rank test was maximal was used. As a result, 4.8 was established as the optimal cut-off point for the VEGF T/N ratio, and tumours with this ratio exceeding 4.8 were defined as showing overexpression of VEGF mRNA. The patients with overexpression of VEGF mRNA demonstrated poorer survival than the patients without overexpression of VEGF mRNA, by both the log-rank test ($P < 0.001$) and the Wilcoxon test ($P = 0.0002$)

### Correlation among VEGF mRNA expression, clinicopathological variables and survival

The correlation between VEGF mRNA expression and clinicopathological variables was analysed statistically using the unpaired *t*-test for comparisons of two groups. Almost all of the patients were followed for approximately 3 years after surgery, and patients' outcome was ascertained in 56 out of 60 cases. Survival was calculated from the date of operation to the date of death or of the last follow-up, and the median follow-up term for the patients without cancer death was 30.1 months. Survival rate was estimated by the Kaplan–Meier method, and statistical significance was compared using the log-rank test and the Wilcoxon test. Univariate and multivariate analyses were performed using Cox's regression model to determine the actual role of VEGF in patient prognosis.

To determine the optimal cut-off point of the VEGF T/N ratio, an analytical method previously reported was used (Miller and Siegmund, 1982). When the VEGF T/N ratio was higher than 4.8, for which the $\chi^2$ value of the log-rank test was maximal, we defined such a tumour as showing overexpression of VEGF mRNA. JMP-3.1.5 software (SAS Institute, Cary, NC, USA) and Stat View-J 4.5 software (Abacus Concepts, Berkeley, CA, USA) were used for the analyses, and $P < 0.05$ was considered to be statistically significant.

**Table 2** Cox's multivariate proportional hazard model for survival

| Variable | Univariate analysis | | | Multivariate analysis | | |
|---|---|---|---|---|---|---|
| | Risk ratio | 95% CI[a] | *P* | Risk ratio | 95% CI[a] | *P* |
| The VEGF T/N ratio (≤ 4.8, >4.8) | 2.49 | 1.61–3.85 | < 0.0001[c] | 1.94 | 1.23–3.06 | 0.005[c] |
| Depth of tumour infiltration (≤pm, >pm) | 3.55 | 1.30–9.72 | 0.013[c] | 2.54 | 0.88–7.30 | 0.084 |
| Maximum diameter (≤5.0 cm, >5.0 cm) | 0.75 | 0.49–1.16 | 0.195 | 0.81 | 0.52–1.26 | 0.350 |
| Tumour differentiation (well, moderate) | 1.88 | 1.02–3.47 | 0.043[c] | 1.21 | 0.63–2.30 | 0.565 |

[a]CI, confidence interval. [c]*P* value < 0.05 was considered to indicate statistical significance.

### RESULTS

### VEGF mRNA expression in colorectal cancer

Five VEGF mRNA splicing variants, which encoded 121, 145 (Poltorak et al, 1997), 165, 189 and 206 (Houck et al, 1991) amino acids respectively were produced from a single gene as a result of alternative splicing. The cDNA encoding $VEGF_{165}$, which is a major isoform of this protein, was used for Northern blot analysis. Representative cases of Northern blot hybridization are shown in Figure 1. The expression of 3.7-kb VEGF mRNA transcript was detected in both tumour and adjacent normal mucosa, and the VEGF T/N ratio in these cases ranged from 0.57 to 23.85. Fifty-two of 60 (86.7%) patients had higher expression in tumour than in non-cancerous mucosa, and in eight cases the VEGF T/N ratio was less than 1.

### Association of the VEGF T/N ratio with patient profile, and clinicopathological variables

The association of VEGF mRNA levels with clinicopathological variables is demonstrated in Table 1. No significant correlation was observed between the levels of VEGF T/N ratio and sex, age, location, size, tumour differentiation, lymphatic involvement or vascular involvement. The VEGF T/N ratio in Dukes' D cases was significantly higher than those in Dukes' A ($P = 0.019$), Dukes' B ($P = 0.018$) and Dukes' C ($P = 0.015$) cases, but no difference was observed among Dukes' A, B and C cases. This ratio was significantly correlated with the depth of tumour infiltration ($P = 0.046$). Furthermore, VEGF mRNA expression was significantly higher in tumours with lymph node metastasis (5.18 ± 5.22) than in those without (2.61 ± 3.75, $P = 0.036$). The mean ± s.d. of VEGF gene expression in cases with synchronous liver metastasis (8.39 ± 7.48) was significantly higher than in cases without (2.57 ± 1.98, $P < 0.0001$).

### Patient prognosis

During the follow-up term, 21 patients died as a result of recurrence (10 of 11 patients with overexpression of VEGF mRNA, and 11 of 45 patients without overexpression of VEGF mRNA). To define the significance of the VEGF T/N ratio as an independent prognostic marker, four variables (the VEGF T/N ratio, tumour size, depth of tumour infiltration and tumour differentiation), which reflect malignant potential of the primary tumour, were selected, and both univariate and multivariate analysis were performed using Cox's regression model (Table 2). According to univariate analysis, overexpression of VEGF mRNA ($P < 0.0001$), depth of tumour infiltration ($P = 0.013$) and tumour differentiation ($P = 0.043$) were associated with poor overall survival of colorectal cancer patients. Multivariate analysis demonstrated that the overall estimated hazard ratio for death in patients with overexpression of VEGF mRNA was 1.94 (95% confidence interval, 1.23–3.06, $P = 0.005$), and that the VEGF T/N ratio was the most significant prognostic marker for overall survival among these variables. Survival rates of these 56 patients with or without overexpression of VEGF mRNA are shown in Figure 2. One-, 2- and 3-year survival rates of the patients without overexpression of VEGF mRNA were 86.7, 77.4 and 74.9% respectively, whereas 1- and 2-year survival rates of the patients with overexpression of VEGF mRNA were 54.5 and 18.2% respectively. The patients with overexpression of VEGF mRNA demonstrated poorer survival than the patients without overexpression of VEGF mRNA by both the log-rank test ($P < 0.001$) and the Wilcoxon test ($P = 0.0002$).

### DISCUSSION

Multistep carcinogenesis in colon cancer is a well-recognized mechanism that was originally proposed by Vogelstein et al (1988). However, no clear explanation has been provided at the molecular level of invasion or metastasis following carcinogenesis. Recent investigations have suggested possible participants in the metastatic potential of cancer cells such as adhesion molecules, proteinases, angiogenic factors, etc. In the present study, we directed our attention to an angiogenic molecule, VEGF, which is the most potent mitogen specific for vascular endothelial cells, mainly because angiogenesis seems to be a prerequisite for the growth of solid tumours.

Many reports have shown a correlation between neovascularization in tumours and VEGF expression, indicating the important role of VEGF in tumour angiogenesis. However, VEGF has some different functions from mitogenic activity for vascular endothelial cells (Gabrilovich et al, 1996; Tsurumi et al, 1997; van der Zee et al, 1997), and we thus attempted to evaluate the association of VEGF mRNA expression with the progression of colorectal cancer apart from its angiogenic role. The reason we chose the VEGF mRNA as a determinant, but not VEGF protein, was that Northern blot analysis is more objective as a quantitative measurement than the immunohistochemical method.

The results here demonstrated that the expression of VEGF mRNA was highest in the Dukes' D class, representing those tumours with either extended lymph node metastasis, liver metastasis or peritoneal dissemination. Actually, higher expression of VEGF mRNA in the primary tumours was observed in the presence of lymph node involvement or hepatic metastasis than in those without. Moreover, VEGF gene expression in the tumour tended to be augmented as the severity of cancer involvement in the vasculolymphatic system increased (data not shown).

VEGF may play a broader role in the biological and pathological characteristics of cancer than was previously thought. The rationale for the participation of VEGF in the metastatic potential of tumours may be as follows. First, an increase in the microvascular density of tumours may increase the probability that cancer cells break loose into the circulatory system. The development of vascular networks possibly leads to increases in lymphocapillary anastomoses, which may contribute to the invasion of cancer cells into the vasculolymphatic system. Second, the ability of tumours to grow at a metastatic site is dependent at least in part on neovascularization (Folkman et al, 1990). Third, VEGF may facilitate invasive potential by degrading the extracellular matrix through plasminogen activation. Namely, VEGF is thought to induce plasminogen activator (PA) in vascular endothelial cells, which leads to the conversion of plasminogen to plasmin (Pepper et al, 1991; Mandriota et al, 1995). Plasmin in turn, activates a latent form of the matrix metalloproteinases (MMPs) (Keski et al, 1992; Montgomery et al, 1993; Baricos et al, 1995). Finally, VEGF inhibits the functional maturation of dendritic cells, which are the most effective antigen-presenting cells in the induction of primary immune responses (Gabrilovich et al, 1996), possibly leading to an immunosuppressive state.

© Cancer Research Campaign 1998

With regard to regulation of VEGF production, hypoxia, glucose deficiency and certain cytokines are supposed to up-regulate VEGF expression (Pertovaara et al, 1994; Li et al, 1995; Shweiki et al, 1995; Cohen et al, 1996). Recently, Kieser et al (1994) reported that mutant p53 up-regulated VEGF production through the activation of protein kinase C. However, we did not obtain a clear correlation between immunohistochemically detected mutation of p53 and mRNA expression of VEGF (the VEGF T/N ratio in p53 mutation negative samples; 4.32 ± 5.64 vs p53 mutation-positive samples; 3.83 ± 4.10), although direct sequencing of the p53 gene was not performed. Furthermore, no significant correlation was seen between the expression of interleukin 1, interleukin 6, transforming growth factor $\beta$ and VEGF (data not shown). Hereafter, more detailed studies are needed to clarify the regulatory mechanism of VEGF expression in colorectal cancer.

In the present study, we examined whether the levels of VEGF mRNA expression in the primary site of colorectal cancer facilitate prediction of patient prognosis or not. When the cut-off point of the VEGF T/N ratio ranged from 2.8 to 12.0, the difference between survivors and non-survivors was statistically significant. To determine the optimal cut-off point of the VEGF T/N ratio, the analytical method introduced by Miller and Siegmund (1982) was used. Consequently, 4.8 was determined to be the optimal cut-off point of the VEGF T/N ratio, and a tumour with this ratio exceeding 4.8 was defined as demonstrating overexpression of VEGF mRNA.

In the multivariate analysis, we selected three variables, depth of tumour infiltration, tumour size and tumour differentiation, other than the VEGF T/N ratio, because all of these variables have been designated as putative prognostic factors based on conventional histopathological studies of primary tumours. This analysis revealed that expression intensity of VEGF mRNA was the most reliable prognostic indicator among these factors.

Thus, we found that VEGF was associated with the progression, and was presumably a significant participant in the metastatic potential of colorectal cancer. These results suggest that the determination of VEGF mRNA expression in colorectal cancer makes it possible to predict post-operative patient prognosis and that the VEGF T/N ratio can be used as an independent prognostic marker in colorectal cancer patients.

### REFERENCES

Asano M, Yukita A, Matsumoto T, Kondo S and Suzuki H (1995) Inhibition of tumor growth and metastasis by an immunoneutralizing monoclonal antibody to human vascular endothelial growth factor/vascular permeability factor$_{121}$. *Cancer Res* **55**: 5296–5301

Baricos WH, Cortez SL, EL-Dahr SS and Schnaper HW (1995) ECM degradation by cultured human mesangial cells is mediated by a PA/plasmin/MMPs-2 cascade. *Kidney Int* **47**: 1039–1047

Berkman RA, Merrill MJ, Reinhold WC, Monacci WT, Saxena A, Clark WC, Robertson JT, Ali IU and Oldfield EH (1993) Expression of the vascular permeability factor/vascular endothelial factor growth gene in central nervous system neoplasms. *J Clin Invest* **91**: 153–159

Brown LF, Berse B, Jackman RW, Tognazzi K, Manseau EJ, Senger D and Dvorak HF (1993) Expression of vascular permeability factor (vascular endothelial growth factor) and its receptors in adenocarcinomas of the gastrointestinal tract. *Cancer Res* **53**: 4727–4735

Cohen T, Nahari D, Cerem LW, Neufeld G and Levi BZ (1996) Interleukin 6 induces the expression of vascular endothelial growth factor. *J Biol Chem* **271**: 736–741

Ferrara N and Henzel WJ (1989) Pituitary follicular cells secrete a novel heparin binding growth factor specific for vascular endothelial cells. *Biochem Biophys Res Commun* **161**: 851–858

Ferrara N, Winer J, Burton T, Rowland A, Siegel M, Phillips HS, Terrell T, Keller GA and Levinson AD (1993) Expression of vascular endothelial growth factor does not promote transformation but confers a growth advantage in vivo to chinese hamster ovary cells. *J Clin Invest* **91**: 160–170

Folkman J (1990) What is the evidence that tumors are angiogenesis dependent? *J Natl Cancer Inst* **82**: 4–6

Gabrilovich DI, Chen H, Girgis KR, Cunningham HT, Meny GM, Nadaf S, Kavanaugh D and Carbone DP (1996) Production of vascular endothelial growth factor by human tumors inhibits the functional maturation of dendritic cells. *Nature Med* **2**: 1096–1103

Houck KA, Ferrara N, Weiner J, Cachianes G, Li B and Leung DW (1991) The Vascular endothelial growth factor family: identification of a fourth molecular species and characterization of alternative splicing of RNA. *Mol Endocrinol* **5**: 1806–1814

Inoue K, Ozeki Y, Suganuma T, Sugiura Y and Tanaka S (1997) Vascular endothelial growth factor expression in primary esophageal squamous cell carcinoma. *Cancer* **79**: 206–213

Keski OJ, Lohi J, Tuuttila A, Tryggvason K and Vartio T (1992) Proteolytic processing of the 72,000-Da type IV collagenase by urokinase plasminogen activator. *Exp Cell Res* **202**: 471–476

Kieser A, Weich HA, Brandner G, Marme D and Kolch W (1994) Mutant p53 potentiates protein kinase C induction of vascular endothelial growth factor expression. *Oncogene* **9**: 963–969

Kondo S, Asano M and Suzuki H (1993) Significance of vascular endothelial growth factor/vascular permeability factor for solid tumor growth, and its inhibition by the antibody. *Biochem Biophys Res Commun* **181**: 902–906

Kuribayashi K, Hikata M, Miyamoto C and Furuichi Y (1988) A rapid and efficient purification of poly(A)$^{+}$-mRNA by oligo(dT)-Latex. *Nucleic Acids Res Symp Series* **19**: 61–64

Li J, Perrella MA, Tsai JC, Yet SF, Hsieh CM, Yoshizumi M, Patterson C, Endege WO, Zhou F and Lee ME (1995) Induction of vascular endothelial growth factor gene expression by interleukin-1 beta in rat aortic smooth muscle cell. *J Biol Chem* **270**: 308–312

Maeda K, Chung Y, Ogawa Y, Takatsuka S, Kang SM, Ogawa M, Sawada T and Sowa M (1996) Prognostic value of vascular endothelial growth factor expression in gastric carcinoma. *Cancer* **77**: 858–863

Mandriota SJ, Seghezzi G, Vassalli JD, Ferrara N, Wasi S, Mazzieri R, Mignatti P and Pepper MS (1995) Vascular endothelial growth factor increases urokinase receptor expression in vascular endothelial cells. *J Biol Chem* **270**: 9709–9716

Marchuk D, Drumm M, Saulino A and Collins FS (1991) Construction of T-vectors, a rapid and general system for direct cloning of unmodified PCR products. *Nucleic Acids Res* **19**: 1154

Mattern J, Koomagi R and Volm M (1996) Association of vascular endothelial growth factor expression with intratumoral microvessel density and tumour cell proliferation in human epidermoid lung carcinoma. *Br J Cancer* **73**: 931–934

Miller R and Siegmund D (1982) Maximally selected chi square statistics. *Biometrics* **38**: 1011–1016

Mise M, Arii S, Higashitsuji H, Furutani M, Niwano M, Harada T, Ishigami S, Toda Y, Nakayama H, Fukumoto M, Fujita J and Imamura M (1996) Clinical significance of vascular endothelial growth factor and basic fibroblast growth factor gene expression in liver tumor. *Hepatology* **23**: 455–464

Montgomery AMP, DeClerck YA, Langley KE, Reisfield RA and Mueller BM (1993) Melanoma-mediated dissolution of extracellular matrix: contribution of urokinase-dependent and metalloproteinase-dependent proteolytic pathways. *Cancer Res* **53**: 693–700

Pepper MS, Ferrara N, Orci L and Montesano R (1991) Vascular endothelial growth factor (VEGF) induces plasminogen activators and plasminogen activator inhibitor-1 in microvascular endothelial cells. *Biochem Biophys Res Commun* **181**: 902–906

Pertovaara L, Kaipainen A, Mustonen T, Orpana A, Ferrara N, Saksela O and Alitalo K (1994) Vascular endothelial growth factor is induced in response to transforming growth factor-beta in fibroblastic and epithelial cells. *J Biol Chem* **269**: 6271–6274

Poltorak Z, Cohen T, Sivan R, Kandelis Y, Spira G, Vlodavsky I, Keshet E and Neufeld G (1997) VEGF145, a secreted vascular endothelial growth factor isoform that binds to extracellular matrix. *J Biol Chem* **272**: 7151–7158

Sambrook J, Fritsch EF and Maniatis T (1989) *Molecular Cloning: a Laboratory Manual*, 2nd edn. Cold Spring Harbor Laboratory Press: Cold Spring Harbor, NY

Samoto K, Ikezaki K, Ono M, Shono T, Kohno K, Kuwano M and Fukui M (1995) Expression of vascular endothelial growth factor and its possible relation with neovascularization in human brain tumors. *Cancer Res* **55**: 1189–1193

Senger DR, Galli SJ, Dvorak AM, Perruzzi CA, Harvey VS and Dvorak HF (1993) Tumor cells secrete a vascular permeability factor that promotes accumulation of ascites fluid. *Science* **219**: 983–985

© Cancer Research Campaign 1998

Shweiki D. Neeman M. Itin A and Keshet E (1995) Induction of vascular endothelial growth factor expression by hypoxia and by glucose deficiency in multicell spheroids: implication for tumor angiogenesis. *Proc Natl Acad Sci USA* **92**: 768–772

Takahashi A. Sasaki H. Kim SJ. Tobisu K. Kakizoe T. Tsukamoto T. Kumamoto Y. Sugimura T and Terada M (1994) Markedly increased amount of messenger RNAs for vascular endothelial growth factor and placenta growth factor in renal cell carcinoma associated with angiogenesis. *Cancer Res* **54**: 4233–4237

Takahashi Y. Kitadai Y. Bucana CD. Cleary KR and Ellis LM (1995) Expression of vascular endothelial growth factor and its receptors. KDR. correlates with vascularity. metastasis. and proliferation of human colon cancer. *Cancer Res* **55**: 3964–3968

Takahashi Y. Tucker SL. Kitadai Y. Koura AN. Bucana CD. Cleary KR and Ellis LM (1997) Vessel counts and expression of vascular endothelial growth factor as prognostic factors in node-negative colon cancer. *Arch Surg* **132**: 541–546

Tischer E. Mitchell R. Hartman T. Silva M. Gospondarowicz D. Fiddes JC and Abraham JA (1991) The human gene for vascular endothelial growth factor. *J Biol Chem* **266**: 11947–11954

Toi M. Hoshina S. Takayanagi T and Tominaga T (1994) Association of vascular endothelial growth factor expression with tumor angiogenesis and with early relapse in primary breast cancer. *Jpn J Cancer Res* **85**: 1045–1049

Tsurumi Y. Murohara T. Krasinski K. Chen D. Witzenbichler B. Kearney M. Couffinhal T and Isner JM (1997) Reciprocal relation between VEGF and NO in the regulation of endothelial integrity. *Nature Med* **3**: 879–886

van der Zee R. Murohara T. Luo Z. Zollmann F. Passeri J. Lekutat C and Isner JM (1997) Vascular endothelial growth factor (VEGF)/vascular permeability factor (VPF) augments nitric oxide release from quiescent rabbit and human vascular endothelium. *Circulation* **95**: 1030–1037

Vogelstein B. Fearon ER. Hamilton SR. Kern SE. Preisinger AC. Leppert M. Nakamura Y. White R. Smits AM and Bos JL (1988) Genetic alterations during colorectal-tumor development. *N Engl J Med* **319**: 525–532

Warren RS. Yuan H. Matli MR. Gillett NA and Ferrara N (1995) Regulation by vascular endothelial growth factor of human colon cancer tumorigenesis in a mouse model of experimental liver metastasis. *J Clin Invest* **95**: 1789–1797

© Cancer Research Campaign 1998


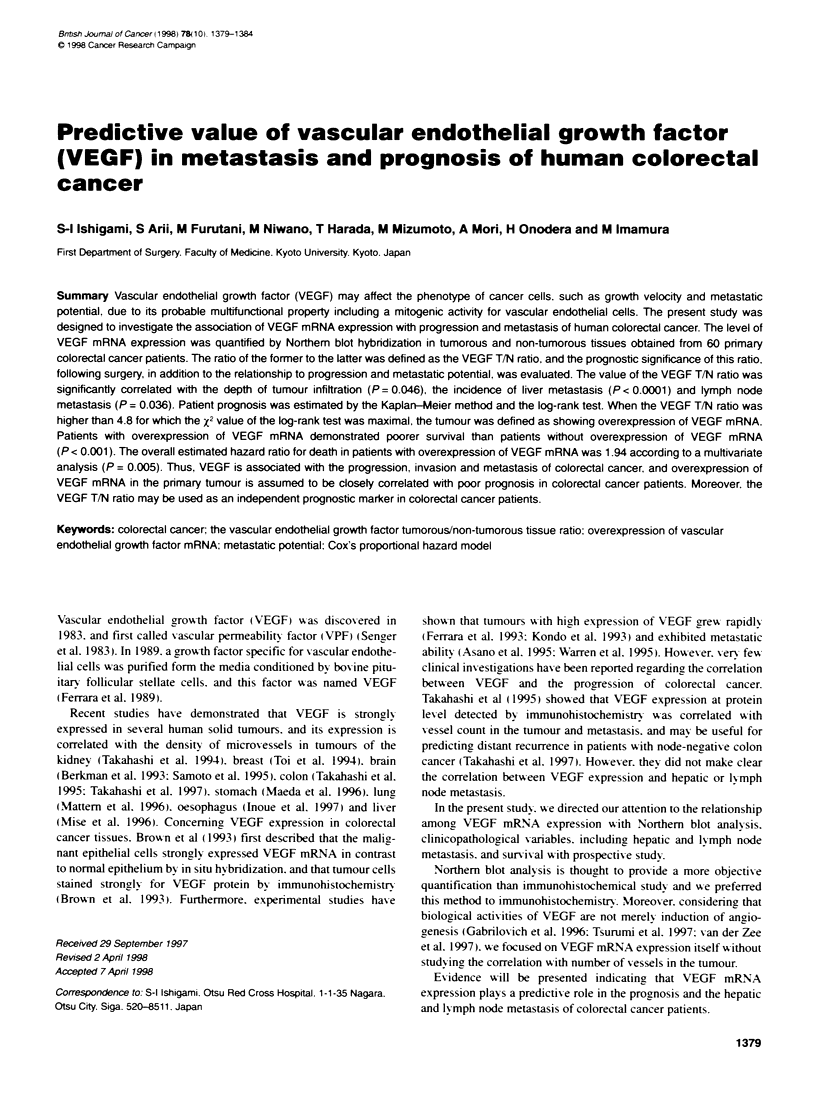

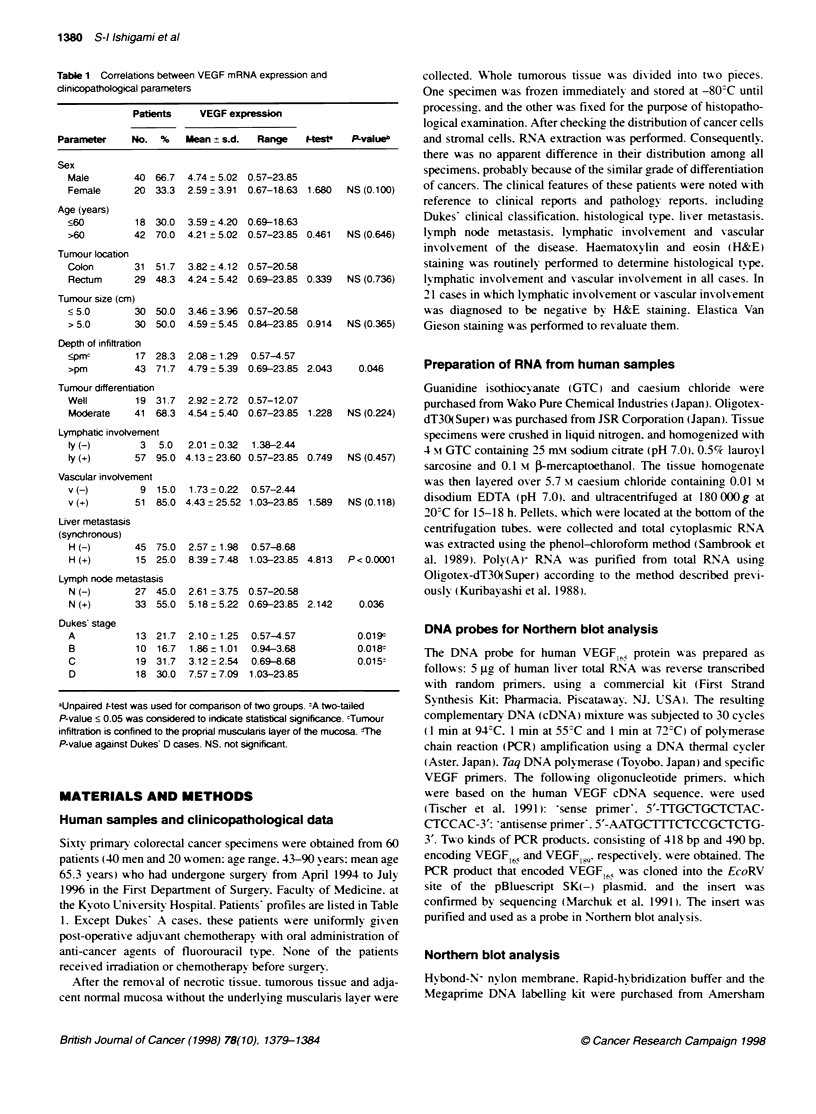

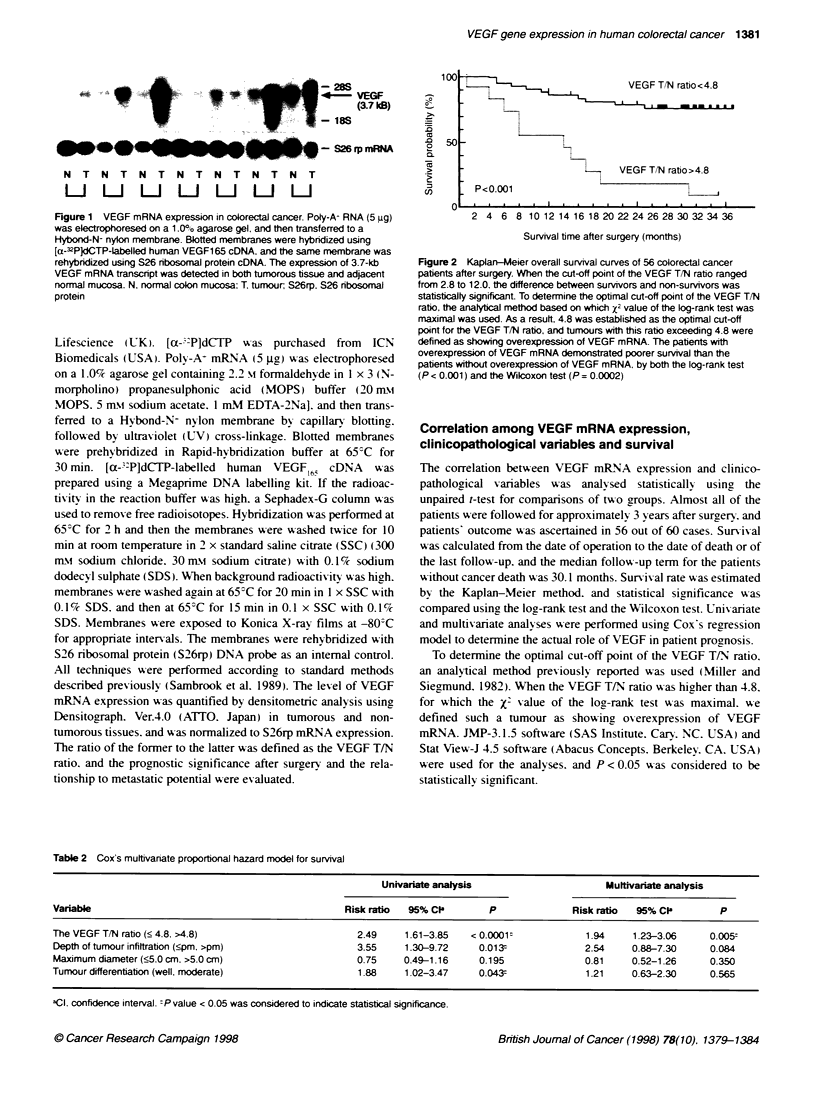

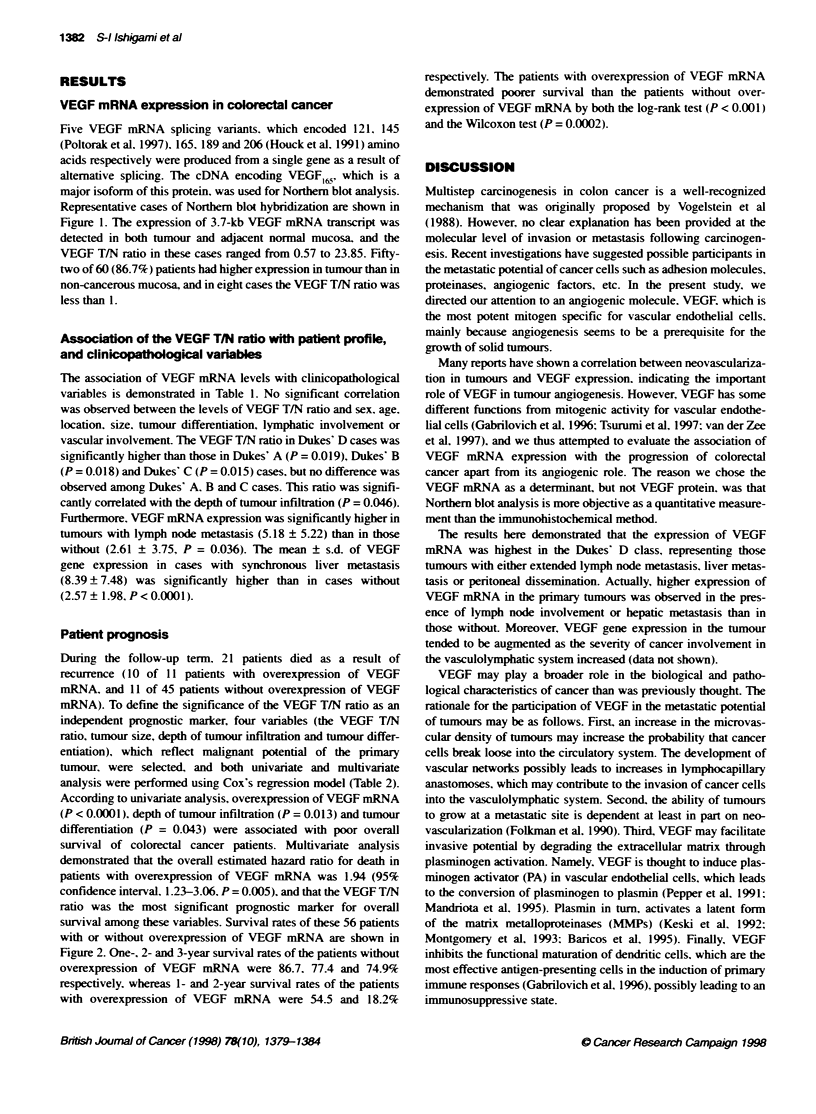

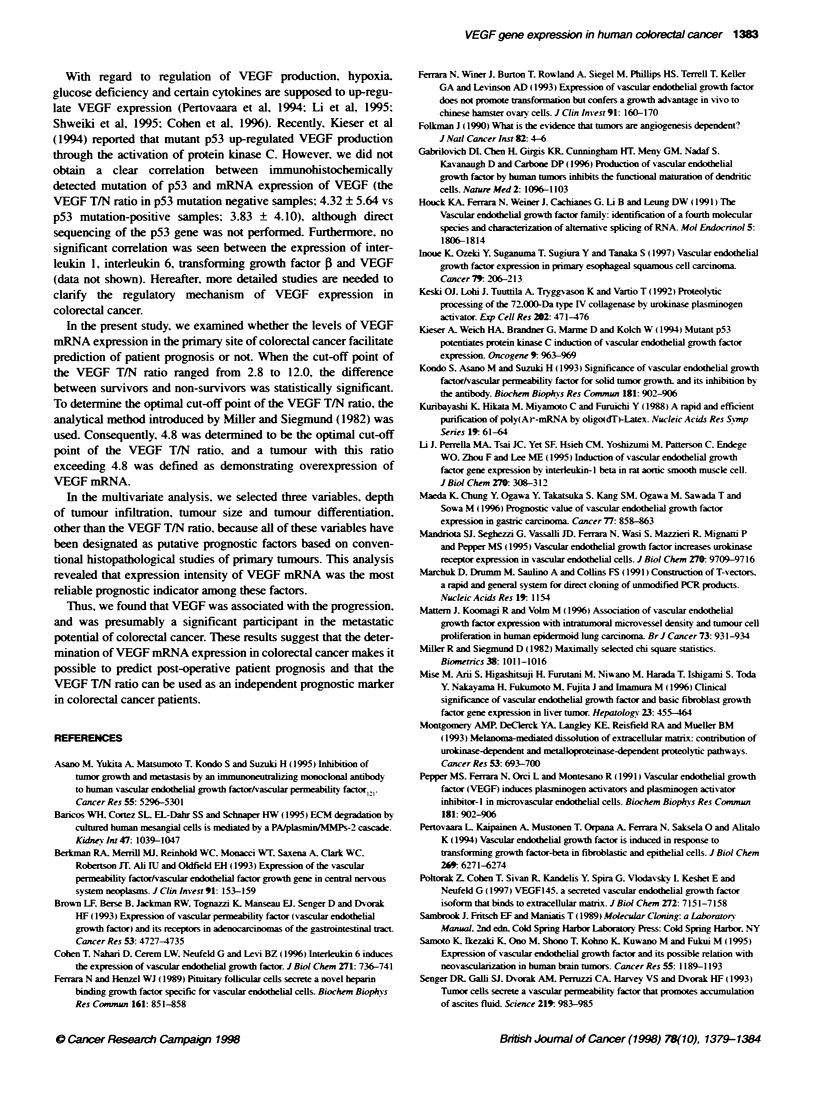

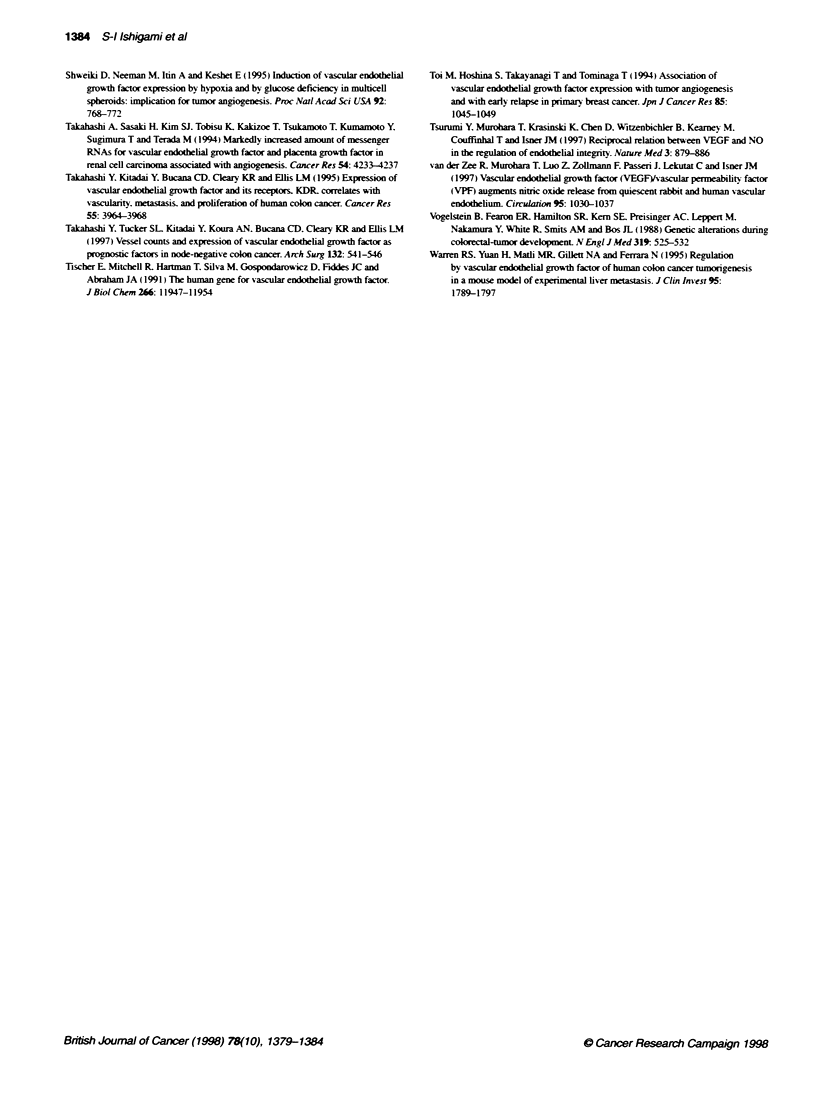


## References

[OCR_00565] Asano M., Yukita A., Matsumoto T., Kondo S., Suzuki H. (1995). Inhibition of tumor growth and metastasis by an immunoneutralizing monoclonal antibody to human vascular endothelial growth factor/vascular permeability factor121.. Cancer Res.

[OCR_00569] Baricos W. H., Cortez S. L., el-Dahr S. S., Schnaper H. W. (1995). ECM degradation by cultured human mesangial cells is mediated by a PA/plasmin/MMP-2 cascade.. Kidney Int.

[OCR_00574] Berkman R. A., Merrill M. J., Reinhold W. C., Monacci W. T., Saxena A., Clark W. C., Robertson J. T., Ali I. U., Oldfield E. H. (1993). Expression of the vascular permeability factor/vascular endothelial growth factor gene in central nervous system neoplasms.. J Clin Invest.

[OCR_00585] Brown L. F., Berse B., Jackman R. W., Tognazzi K., Manseau E. J., Senger D. R., Dvorak H. F. (1993). Expression of vascular permeability factor (vascular endothelial growth factor) and its receptors in adenocarcinomas of the gastrointestinal tract.. Cancer Res.

[OCR_00588] Cohen T., Nahari D., Cerem L. W., Neufeld G., Levi B. Z. (1996). Interleukin 6 induces the expression of vascular endothelial growth factor.. J Biol Chem.

[OCR_00591] Ferrara N., Henzel W. J. (1989). Pituitary follicular cells secrete a novel heparin-binding growth factor specific for vascular endothelial cells.. Biochem Biophys Res Commun.

[OCR_00596] Ferrara N., Winer J., Burton T., Rowland A., Siegel M., Phillips H. S., Terrell T., Keller G. A., Levinson A. D. (1993). Expression of vascular endothelial growth factor does not promote transformation but confers a growth advantage in vivo to Chinese hamster ovary cells.. J Clin Invest.

[OCR_00602] Folkman J. (1990). What is the evidence that tumors are angiogenesis dependent?. J Natl Cancer Inst.

[OCR_00608] Gabrilovich D. I., Chen H. L., Girgis K. R., Cunningham H. T., Meny G. M., Nadaf S., Kavanaugh D., Carbone D. P. (1996). Production of vascular endothelial growth factor by human tumors inhibits the functional maturation of dendritic cells.. Nat Med.

[OCR_00613] Houck K. A., Ferrara N., Winer J., Cachianes G., Li B., Leung D. W. (1991). The vascular endothelial growth factor family: identification of a fourth molecular species and characterization of alternative splicing of RNA.. Mol Endocrinol.

[OCR_00621] Inoue K., Ozeki Y., Suganuma T., Sugiura Y., Tanaka S. (1997). Vascular endothelial growth factor expression in primary esophageal squamous cell carcinoma. Association with angiogenesis and tumor progression.. Cancer.

[OCR_00626] Keski-Oja J., Lohi J., Tuuttila A., Tryggvason K., Vartio T. (1992). Proteolytic processing of the 72,000-Da type IV collagenase by urokinase plasminogen activator.. Exp Cell Res.

[OCR_00631] Kieser A., Weich H. A., Brandner G., Marmé D., Kolch W. (1994). Mutant p53 potentiates protein kinase C induction of vascular endothelial growth factor expression.. Oncogene.

[OCR_00639] Kuribayashi K., Hikata M., Hiraoka O., Miyamoto C., Furuichi Y. (1988). A rapid and efficient purification of poly(A)-mRNA by oligo(dT)30-Latex.. Nucleic Acids Symp Ser.

[OCR_00644] Li J., Perrella M. A., Tsai J. C., Yet S. F., Hsieh C. M., Yoshizumi M., Patterson C., Endege W. O., Zhou F., Lee M. E. (1995). Induction of vascular endothelial growth factor gene expression by interleukin-1 beta in rat aortic smooth muscle cells.. J Biol Chem.

[OCR_00653] Maeda K., Chung Y. S., Ogawa Y., Takatsuka S., Kang S. M., Ogawa M., Sawada T., Sowa M. (1996). Prognostic value of vascular endothelial growth factor expression in gastric carcinoma.. Cancer.

[OCR_00656] Mandriota S. J., Seghezzi G., Vassalli J. D., Ferrara N., Wasi S., Mazzieri R., Mignatti P., Pepper M. S. (1995). Vascular endothelial growth factor increases urokinase receptor expression in vascular endothelial cells.. J Biol Chem.

[OCR_00660] Marchuk D., Drumm M., Saulino A., Collins F. S. (1991). Construction of T-vectors, a rapid and general system for direct cloning of unmodified PCR products.. Nucleic Acids Res.

[OCR_00665] Mattern J., Koomägi R., Volm M. (1996). Association of vascular endothelial growth factor expression with intratumoral microvessel density and tumour cell proliferation in human epidermoid lung carcinoma.. Br J Cancer.

[OCR_00677] Mise M., Arii S., Higashituji H., Furutani M., Niwano M., Harada T., Ishigami S., Toda Y., Nakayama H., Fukumoto M. (1996). Clinical significance of vascular endothelial growth factor and basic fibroblast growth factor gene expression in liver tumor.. Hepatology.

[OCR_00682] Montgomery A. M., De Clerck Y. A., Langley K. E., Reisfeld R. A., Mueller B. M. (1993). Melanoma-mediated dissolution of extracellular matrix: contribution of urokinase-dependent and metalloproteinase-dependent proteolytic pathways.. Cancer Res.

[OCR_00686] Pepper M. S., Ferrara N., Orci L., Montesano R. (1991). Vascular endothelial growth factor (VEGF) induces plasminogen activators and plasminogen activator inhibitor-1 in microvascular endothelial cells.. Biochem Biophys Res Commun.

[OCR_00695] Pertovaara L., Kaipainen A., Mustonen T., Orpana A., Ferrara N., Saksela O., Alitalo K. (1994). Vascular endothelial growth factor is induced in response to transforming growth factor-beta in fibroblastic and epithelial cells.. J Biol Chem.

[OCR_00702] Poltorak Z., Cohen T., Sivan R., Kandelis Y., Spira G., Vlodavsky I., Keshet E., Neufeld G. (1997). VEGF145, a secreted vascular endothelial growth factor isoform that binds to extracellular matrix.. J Biol Chem.

[OCR_00708] Samoto K., Ikezaki K., Ono M., Shono T., Kohno K., Kuwano M., Fukui M. (1995). Expression of vascular endothelial growth factor and its possible relation with neovascularization in human brain tumors.. Cancer Res.

[OCR_00715] Senger D. R., Galli S. J., Dvorak A. M., Perruzzi C. A., Harvey V. S., Dvorak H. F. (1983). Tumor cells secrete a vascular permeability factor that promotes accumulation of ascites fluid.. Science.

[OCR_00722] Shweiki D., Neeman M., Itin A., Keshet E. (1995). Induction of vascular endothelial growth factor expression by hypoxia and by glucose deficiency in multicell spheroids: implications for tumor angiogenesis.. Proc Natl Acad Sci U S A.

[OCR_00730] Takahashi A., Sasaki H., Kim S. J., Tobisu K., Kakizoe T., Tsukamoto T., Kumamoto Y., Sugimura T., Terada M. (1994). Markedly increased amounts of messenger RNAs for vascular endothelial growth factor and placenta growth factor in renal cell carcinoma associated with angiogenesis.. Cancer Res.

[OCR_00734] Takahashi Y., Kitadai Y., Bucana C. D., Cleary K. R., Ellis L. M. (1995). Expression of vascular endothelial growth factor and its receptor, KDR, correlates with vascularity, metastasis, and proliferation of human colon cancer.. Cancer Res.

[OCR_00741] Takahashi Y., Tucker S. L., Kitadai Y., Koura A. N., Bucana C. D., Cleary K. R., Ellis L. M. (1997). Vessel counts and expression of vascular endothelial growth factor as prognostic factors in node-negative colon cancer.. Arch Surg.

[OCR_00747] Tischer E., Mitchell R., Hartman T., Silva M., Gospodarowicz D., Fiddes J. C., Abraham J. A. (1991). The human gene for vascular endothelial growth factor. Multiple protein forms are encoded through alternative exon splicing.. J Biol Chem.

[OCR_00750] Toi M., Hoshina S., Takayanagi T., Tominaga T. (1994). Association of vascular endothelial growth factor expression with tumor angiogenesis and with early relapse in primary breast cancer.. Jpn J Cancer Res.

[OCR_00758] Tsurumi Y., Murohara T., Krasinski K., Chen D., Witzenbichler B., Kearney M., Couffinhal T., Isner J. M. (1997). Reciprocal relation between VEGF and NO in the regulation of endothelial integrity.. Nat Med.

[OCR_00767] Vogelstein B., Fearon E. R., Hamilton S. R., Kern S. E., Preisinger A. C., Leppert M., Nakamura Y., White R., Smits A. M., Bos J. L. (1988). Genetic alterations during colorectal-tumor development.. N Engl J Med.

[OCR_00772] Warren R. S., Yuan H., Matli M. R., Gillett N. A., Ferrara N. (1995). Regulation by vascular endothelial growth factor of human colon cancer tumorigenesis in a mouse model of experimental liver metastasis.. J Clin Invest.

[OCR_00763] van der Zee R., Murohara T., Luo Z., Zollmann F., Passeri J., Lekutat C., Isner J. M. (1997). Vascular endothelial growth factor/vascular permeability factor augments nitric oxide release from quiescent rabbit and human vascular endothelium.. Circulation.

